# Quantitative evaluation of ^67^Ga-citrate scintigraphy in the management of nephritis

**DOI:** 10.1038/s41598-024-66823-2

**Published:** 2024-07-15

**Authors:** Noritake Matsuda, Hideki Otsuka, Ryosuke Kasai, Tamaki Otani, Leah Anne Christine Locsin Bollos, Shota Azane, Yamato Kunikane, Yoichi Otomi, Yuya Ueki, Mana Okabe, Masafumi Amano, Masanori Tamaki, Shu Wakino, Shoichiro Takao, Masafumi Harada

**Affiliations:** 1 Department of Radiology, Tokushima University Hospital, Kuramoto-cho 2-50-1, Tokushima, 770-8503 Japan; 2 Department of Medical Imaging/Nuclear Medicine, Tokushima University Graduate School of Biomedical Sciences, Kuramoto-cho 3-18-15, Tokushima, Tokushima 770-8503 Japan; 3 Advance Radiation Research, Education and Management Center, Tokushima University, Kuramoto-cho 3-18-15, Tokushima, Tokushima 770-8503 Japan; 4 Tokushima University Graduate School of Biomedical Sciences, Kuramoto-cho 3-18-15, Tokushima, Tokushima 770-8503 Japan; 5 Department of Radiology and Radiation Oncology, Tokushima University Graduate School of Biomedical Sciences, Kuramoto-cho 2-50-1, Tokushima, 770-8503 Japan; 6 Radiology Service, Shiga University of Medical Science Hospital, Seta Tsukinowacho, Otsu-shi, Shiga, 520-2192 Japan; 7 Department of Nephrology, Tokushima University Graduate School of Biomedical Sciences, Kuramoto-cho 2-50-1, Tokushima, 770-8503 Japan

**Keywords:** ^67^Ga-citrate scintigraphy, Interstitial nephritis, Standardized uptake value, Active nephritis volume, Total nephritis uptake, Molecular biology, Biomarkers, Diseases, Medical research, Molecular medicine, Nephrology

## Abstract

In ^67^Ga-citrate scintigraphy (Ga-S), visual assessment is used by evaluating renal-uptake comparison with liver and spine and is simple and objective. We adopted the standardized uptake value (SUV) for ^67^Ga-citrate and proposed two quantitative indices, active nephritis volume (ANV) and total nephritis uptake (TNU). This study clarified the utility of new Ga-S-based quantitative indices in nephritis management. Before SUV measurement, the Becquerel calibration factor of ^67^Ga-citrate was obtained using a phantom experiment. Seventy patients who underwent SPECT/CT imaging were studied. SUV, ANV, and TNU were calculated using a quantitative analysis software for bone SPECT. SUV_mean_, ANV, and TNU were analyzed using the (1) threshold method (set 40%) and constant-value method for (2) vertebral SUV_max_, and (3) vertebral SUV_mean_. ROC analysis was used to evaluate SUV, ANV, and TNU diagnostic abilities to distinguish nephritis presence and absence as well as interstitial nephritis (IN) and non-IN. The area under the curve (AUC) for nephritis presence or absence had a good value (0.80) for SUV_mean_ (1), ANV (3), and TNU (3). The AUC for differentiation between IN and non-IN groups had a good value (0.80) for SUV_mean_ (1). Thus, the new Ga-S-based quantitative indices were useful to evaluate nephritis and distinguish IN and non-IN.

## Introduction

^67^Ga-citrate scintigraphy (Ga-S) plays an important role in the management of certain types of tumors and inflammation, such as sarcoidosis, malignant lymphoma, melanoma, and inflammatory disease. Ga-S is scanned 48 h after radiotracer injection. Physiological uptake can be observed in the lacrimal glands, liver, and vertebral bodies. Except for physiological accumulation, gallium excretion from the kidney into the urine is almost complete within 24 h after injection, and the kidney shows almost the same uptake as the background and could not be visualized after 48 h of scanning. Renal diseases have been suggested when renal uptake is identified^1,2^. Nephritis is a major indication of Ga-S. Inflammatory diseases of the renal parenchyma are broadly classified as interstitial nephritis (IN) and glomerulonephritis. ^67^Ga scintigraphy may be useful for patients with a clinical suspicion of acute interstitial nephritis, especially those who are unable to undergo kidney biopsy^3^. The usefulness of gallium scintigraphy for IN has been previously reported^4^. Visual assessment is widely used to evaluate renal uptake, which is graded by comparing the degree of uptake to that of the liver and vertebral bodies using planar and SPECT images^2,5,6^. Visual grading is simple but objective, less quantitative, and problematic because it is affected by the degree of physiological accumulation in the liver and spine. To date, there have been very few reports on the quantitative analysis of Ga-S^7^.

The standardized uptake value (SUV) was first introduced for positron emission tomography (PET) and is the most commonly used quantitative index for PET; however, it is rarely used for SPECT. Thus, quantitative evaluation using the SUV is an advantage of PET over SPECT. A software program that can calculate the SUV, perform quantitative analysis of bone SPECT/CT, and evaluate uptake was developed and implemented^8,9^. We previously reported quantitative methods and new indices for SPECT/CT examinations^10–12^. We adopted the SUV for ^67^Ga-citrate and proposed two quantitative indices, active nephritis volume (ANV) and total nephritis uptake (TNU), corresponding to the volume of renal inflammation. This study aimed to examine the relationship between conventional visual assessment and quantitative indices and to clarify the utility of new Ga-S-based quantitative indices in the management of nephritis.

## Methods

This single-center retrospective study was performed at our institution after obtaining approval from the Ethics Committee of Tokushima University Hospital (approved number 4030-3), and the Declaration of Helsinki was followed by all the participant researchers. The requirement for written informed consent was waived. The information disclosure document for this study is publicly available on Tokushima University Hospital website.

The phantom and clinical studies were performed using a hybrid SPECT/CT system (Symbia T16, Siemens, Germany).

### Phantom study

Before the SUV measurement, a phantom experiment was performed to calculate the Becquerel calibration factor (BCF) for converting the counts of the reformatted SPECT images to the radioactivity concentration. A cylindrical phantom (inner diameter, 16 cm; length, 15 cm; volume, 3016 ml; Sangyo Kagaku, Tokyo, Japan) was prepared using water and ^67^Ga-citrate at known concentrations. The phantom was scanned for 15 min, and the data were reconstructed according to the clinical ^67^Ga-citrate SPECT/CT protocol (Table 1). BCF acquired using the bone SPECT analysis software, GI-BONE (AZE Corp., Tokyo, Japan), was used to calculate the SUV in this study.

**Table 1:** Image processing.

SPECT/CT scanner	Symbia T16 (Siemens)
RI	^67^ Ga-citrate
Colimator	LMEGP
keV	93 keV ± 20%
	185 keV ± 15%
Matrix	128 × 128
Pixel size	3.9 mm
Magnification power	1.23
Image processing	Continuous mode
Detector distance	Automatic proximity shooting
Collection time	30 s × 30
Rotation	180°
Attenuation correction	CTAC
Scatter correction	–

### Patient study

Seventy nephrology patients who underwent ^67^Ga-citrate scintigraphy at our hospital between January 2015 and September 2022 were studied in this retrospective study (IN, n = 36; non-IN, n = 29; unknown, n = 5; men, n = 48; women, n = 22; age, 15–87 years; Table 2). The clinical diagnosis was confirmed by a board-certified nephrologist. Seventeen patients were histologically diagnosed with IN using renal biopsy and nineteen were clinically diagnosed with IN. In every patient, approximately 111 MBq of ^67^Ga-citrate was injected intravenously, a whole-body planar image was obtained, and SPECT/CT was performed 48 h after injection. Computed tomography data were used for attenuation correction and to obtain anatomical information.

**Table 2:** Patient characteristics in interstitial nephritis (IN) and non-IN.

Creatinine (SD)	Interstitial nephritis		Non-interstitial nephritis		*p* value
Number of patient	36		29		–
Number of kidney	71		57		–
Men/women	25/11		20/9		0.97
Age: mean (range)	61.5 (15–87)		64.4 (28–85)		0.49
Body weight: mean (SD)	60.4 (16.8)		60.2 (17.9)		0.97
Creatinine (SD)	3.1 (2.3)		3.1 (2.2)		0.98
	Interstitial nephritis	26	Nephrosclerosis	9	
	Interstitial nephritis + other diseases	10	Prerenal renal failure	3	
			Diabetic nephropathy	2	
			Diabetic kidney disease	2	
			Sarcoidosis	2	
			Membranous nephropathy	2	
			Others	9	

In Table, Student’s t-test was used to compare patient characteristics, such as sex, age, and body weight between IN and non-IN subjects. There were no significant differences in sex, age, weight, and creatinine between IN and non-IN subjects.

### Visual evaluation

**Figure 1:**
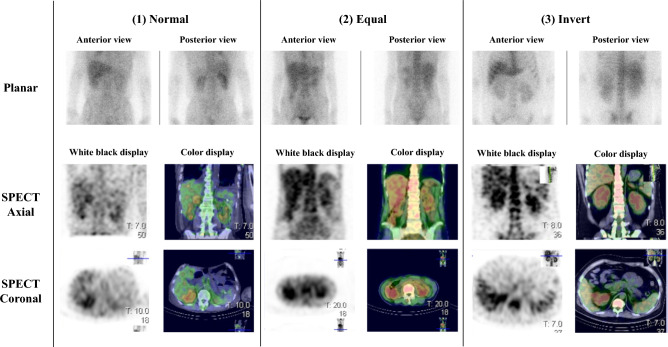
Uptake pattern. The anterior and posterior planar images and SPECT axial and coronal images in black and white and in color for each uptake pattern are shown. (1) *Normal* Normal uptake pattern with higher liver uptake than vertebral uptake. (2) *Equal* Abnormal uptake pattern with liver uptake equal to vertebral uptake. (3) *Inverted* Abnormal uptake pattern with vertebral uptake higher than liver uptake.

Classification of the participants into three groups was performed by two certified nuclear medicine specialists based on the uptake pattern (Fig. 1).

1. *Normal* Normal uptake pattern with higher liver uptake than vertebral uptake.
2. *Equal* Abnormal uptake pattern with liver uptake equal to vertebral uptake.
3. *Inverted* an abnormal uptake pattern with higher vertebral uptake than liver uptake.

The 6-score grading of renal uptake was performed by two board-certified nuclear medicine specialists^2^.

- Grade 0: No renal uptake;
- Grade 1: < spine;
- Grade 2: = spine;
- Grade 3: spine < , < liver;
- Grade 4: = liver;
- Grade 5: > liver.

### Quantitative indices

#### SUV

The radiation count was converted to radioactivity using the BCF calculated using the quantification software program for bone SPECT (GI-BONE; AZE Corp., Tokyo, Japan). It was calculated using the formula:

$$ {\text{Radioactivity of the region}} \,\left[ {{\text{Bq}}} \right] = {\text{radiation count of the region}}\, \left[ {{\text{cps}}} \right] \times {\text{BCF}}\, \left[ {\frac{{{\text{Bq}}}}{{{\text{cps}}}}} \right]. $$

SUV was calculated using the formula:

$$ {\text{SUV}} = \frac{{ {\text{the mean volume of interest}}\, \left( {{\text{VOI}}} \right)\; {\text{activity}}\, \left[ {\text{Bq/ml}} \right]}}{{{\text{injected dose}} \,\left[ {{\text{Bq}}} \right] / {\text{body weight}}\, \left[ {\text{g}} \right]}} = \frac{{{\text{total count of VOI}}\, \left[ {{\text{cps}}} \right] \times {\text{BCF}}\, \left[ {\text{Bq/cps}} \right]/ {\text{the volume of VOI }}\,\left[ {{\text{ml}}} \right]}}{{{\text{injected dose}}\, \left[ {{\text{Bq}}} \right]/ {\text{body weight}}\, \left[ {\text{g}} \right]}}. $$

The SUV of the kidneys, liver, and vertebrae were measured separately using the previous BCF. The SUV_max_, SUV_peak_, and SUV_mean_ values of the kidneys, liver, and vertebrae were calculated.

**Figure 2:**
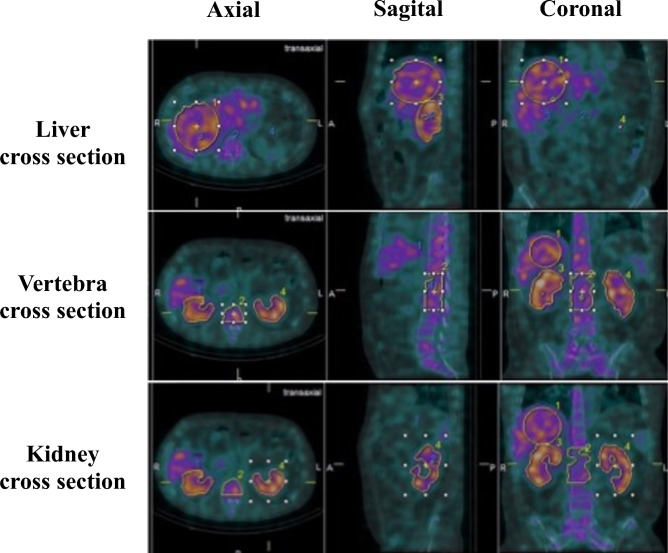
Setting the volume of interest for the liver, vertebrae, and kidneys. In the figure, yellow frame 1 indicates the liver, 2 indicates the vertebral body, 3 indicates the right kidney, and 4 indicates the left kidney. The entire kidney was set as the VOI to avoid including other organs. The VOI of the two vertebrae was set at the kidney level. Intense focal uptake was suggestive of vertebral degeneration; osteophytes were excluded.

There are restrictions on the VOI shape settings of the quantitative analysis software used in this study (GI-BONE). The VOI shape cannot be set to an arbitrary shape; it must be set to one of the following shapes: rectangular parallelepiped, cylinder, or sphere. After setting the VOI for the target organ in either shape, the VOI setting is completed by setting the threshold. A sphere was used to set the VOI shape of the liver and kidney, and a rectangular parallelepiped was used to set the VOI shape of the vertebral body. To set the VOI of the liver and vertebrae SUV_mean_, 40% of the SUV_max_ of the VOI, which is the default value of GI-BONE, was used as the threshold. The renal SUV_mean_ was calculated using three methods. The entire kidney was set as the VOI to avoid including other organs. The VOI of the two vertebrae was set at the kidney level. Intense focal uptake was suggestive of vertebral degeneration and osteophytes were excluded (Fig. 2). Two quantitative indices were proposed for ^67^Ga-citrate: ANV and TNU.

### Active nephritis volume

**Figure 3:**
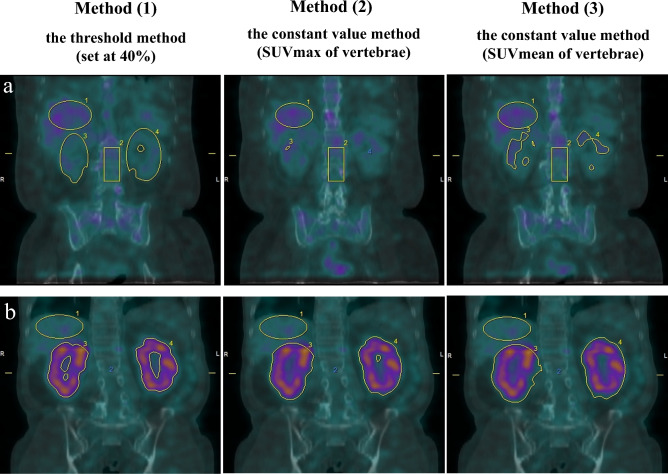
Three analysis methods for renal SUV_mean_ and ANV in grade 2 (**a**), grade 5 (**b**). (**a**) Grade 2: 40% = 0.92; vertebrae SUV_max_ = 2.3; vertebrae SUV_mean_ = 1.7. (**b**) Grade 5: 40% = 0.64; vertebrae SUV_max_ = 1.6; vertebrae SUV_mean_ = 1.2. In the case shown in Figure, the difference in VOI due to the difference in threshold settings is not a big difference in grade 5, but there is a big difference in grade 2. Lower grades are more susceptible to threshold settings. Method (1): VOI is set wider than the actual renal uptake, and there is a possibility of overestimation. Method (2): The VOI is set narrower than the actual renal uptake, and there is a possibility of underestimation. Method (3): Among these three methods, renal uptake was the most appropriate to set as VOI.

The active nephritis volume (ANV) corresponded to the metabolic tumor volume (MTV) on FDG-PET. This represents the volume of voxels with an SUV exceeding the cut-off value. Renal inflammation was defined as the region exceeding the cutoff value. ANV was analyzed using three methods: (1) the threshold method (set at 40%), (2) the constant value method (SUV_max_ of vertebrae), and (3) the constant value method (SUV_mean_ of vertebrae) (Fig. 3).

### Total nephritis uptake

Total nephritis uptake (TNU) corresponded to total lesion glycolysis (TLG) on FDG-PET. TNU was calculated using the following formula:

$$ {\text{TNU}} = {\text{ANV}} \times {\text{SUVmean}}. $$

### Renal SUV_mean_/vertebrae SUV_mean_

The renal/vertebral SUV_mean_ was calculated by dividing the renal SUV_mean_ by the vertebral SUV_mean_. Renal SUV_mean_ was calculated using each of the three thresholding methods; therefore, it was also calculated for each of the renal/vertebrae SUV_mean_.

The diagnostic abilities of SUV_max_, SUV_peak_, SUV_mean_, ANV, TNU, and renal/vertebral SUV_mean_ ratio for the presence or absence of inflammation in the IN (n = 36) and non-IN groups (n = 29) were assessed using ROC analysis and AUC. The diagnostic abilities of SUV_max_, SUV_peak_, SUV_mean_, ANV, TNU, and renal/vertebral SUV_mean_ for differentiating the IN (n = 26) from the non-IN group (n = 29) were also assessed using ROC analysis and AUC.

### Statistical analysis

**Figure 4:**
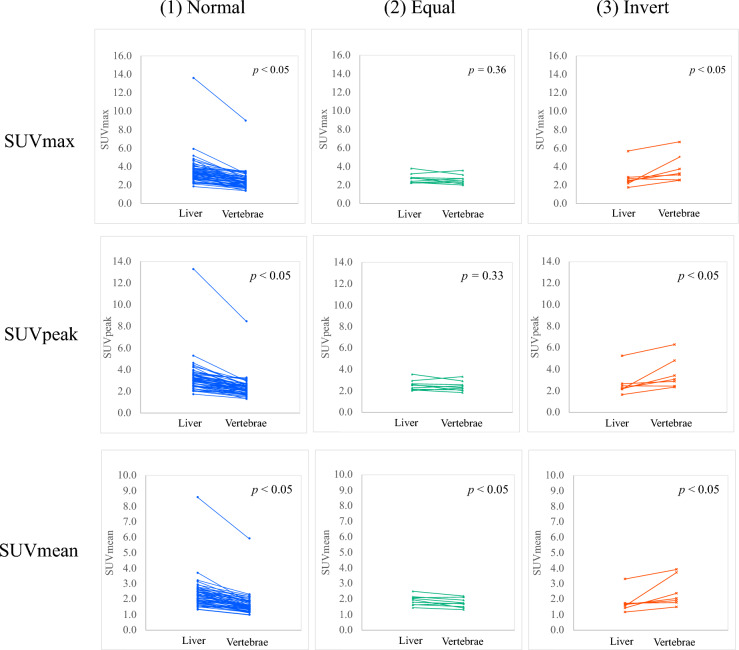
Comparison of SUVs of the liver and vertebrae. In Figure, paired t-test was used to compare liver SUV and vertebrae SUV. In (1) the Normal uptake pattern, liver SUV was significantly higher than vertebrae SUV. In (2) the Equal uptake pattern, there was no significant difference between liver SUVmax and vertebrae SUVmax, liver SUVpeak and vertebrae SUVpeak; liver SUVmean was significantly higher than vertebrae SUVmean. In (3) the Invert uptake pattern, vertebrae SUV was significantly higher than liver SUV.

**Figure 5:**
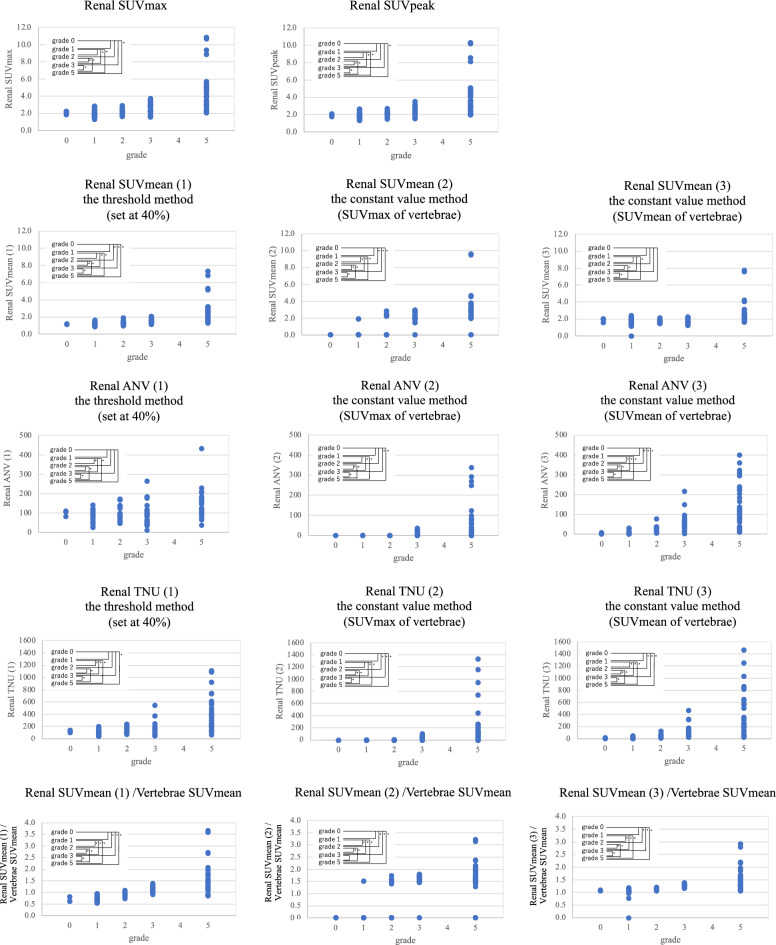
Relationship between the grade and each quantitative index. In Figure, after F test, Student’s t-test or Welch’s t test was used to compare between each set of two grades. *p < 0.05 in comparison between each two grades. Each set of two grades showed statistically significant differences except for grade 0, 1, 2, and 3, and grade 1 and 2 in SUVmax and SUVpeak; grade 0 and 1 and grade 1 and 2 in SUVmean (1); grade 0 and 1 in SUVmean (2); grade 0, 1, 2, 3, and 5, grade 1, 2, and 3, and grade 2 and 3 in SUVmean (3); grade 0, 1, 2, 3, and 5, grade 1 and 3, and grade 2 and 3 in ANV (1); grade 0, 1, and 2 and grade 1 and 2 in ANV (2); grade 0 and 1 in ANV (3); grade 0, 1, 2, and 3, and grade 2 and 3 in TNU (1); grade 0 and 1, grade 1 and 2 in TNU (2); grade 0 and 1 in TNU (3); grade 0 and 1 in SUVmean (1)/vertebrae SUVmean; grade 0 and 1 and grade 3 and 5 in SUVmean (2)/vertebrae SUVmean; and grade 0 and 1 in SUVmean (3)/vertebrae SUVmean.

**Figure 6:**
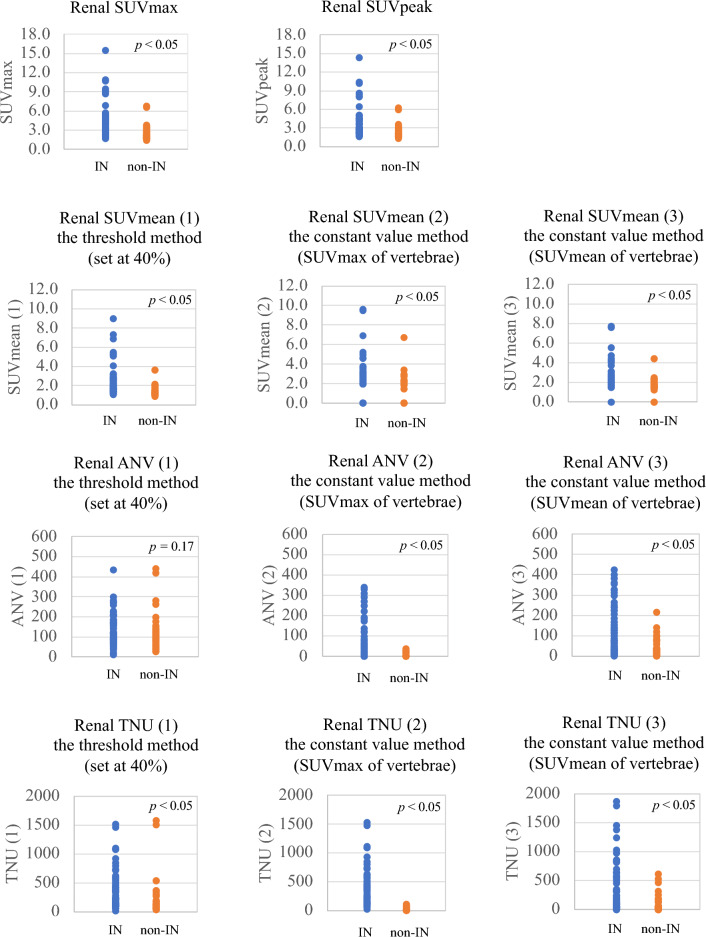
Quantitative indices for each disease. In Figure, after F test, Student’s t-test or Welch’s t test was used to compare IN and non-IN. There were significant differences between IN groups and non-IN groups in all quantitative indicators except ANV (1).

In Fig. 4, the paired t-test was used to compare liver SUV and vertebrae SUV. In Fig. 5, after F test, Student’s t-test or Welch’s t test was used to compare each set of two grades. In Fig. 6, after F test, Student’s t-test or Welch’s t test was used to compare IN and non-IN. In Table 2, Student’s t-test was used to compare patient characteristics, such as sex, age, and body weight between IN and non-IN. In Table 3, one-way analysis of variance was used to compare patient characteristics, such as sex, age, and body weight among the three groups: (1) Normal, (2) Equal, and (3) Invert. In Table 4, one-way analysis of variance was used to compare patient characteristics, such as sex, age, and body weight among the five grades: grade 0, grade 1, grade 2, grade 3, and grade 5. In Table 5, one-way analysis of variance was used to compare each index, including SUVmax, SUVpeak, SUVmean, ANV, TNU, and Renal/vertebrae SUVmean among the three groups: (1) Normal, (2) Equal, and (3) Invert. In Table 6, one-way analysis of variance was used to compare each index, including SUVmax, SUVpeak, SUVmean, ANV, TNU, and Renal/vertebrae SUVmean among the five grades: grade 0, grade 1, grade 2, grade 3, and grade 5. In Table 7, after F test, Student’s t-test or Welch’s t test was used to compare each set of two grades. In Table 8, after F test, Student’s t-test or Welch’s t test was used to compare between each set of two groups: IN, IN + other diseases—non-IN, IN only—non-IN. Statistical significance was set at *p* < 0.05.

**Table 3:** Patient characteristics in uptake patterns.

	(1) Normal	(2) Equal	(3) Invert	Total	*p* value
Number of patient	54	9	7	70	–
Number of kidney	105	18	14	137	–
Men/women	38/16	4/5	6/1	48/22	0.33
Age: mean (range)	63.1 (15–87)	64.4 (31–79)	57.1 (42–68)	62.6 (15–87)	0.82
Body weight: mean (SD)	60.4 (17.0)	51.6 (11.1)	66.7 (20.6)	59.9 (16.9)	0.34
Creatinine (SD)	3.0 (2.3)	3.8 (2.7)	3.0 (1.4)	3.1 (2.3)	0.81

In Table, one-way analysis of variance was used to compare patient characteristics, such as sex, age, and body weight among three groups: (1) Normal, (2) Equal, and (3) Invert. There were no significant differences in sex, age, weight, and creatinine levels among the three uptake patterns.

**Table 4:** Patient characteristics in the grade classification of normal uptake pattern.

	Grade 0	Grade 1	Grade 2	Grade 3	Grade 4	Grade 5	*p* value
Number of patient	2	15	9	11	0	18	–
Number of kidney	3	29	17	22	–	34	–
Men/women	2/0	10/5	5/4	7/4	–	14/4	0.67
Age: mean (range)	56.0 (46–66)	57.5 (15–85)	70.0 (43–83)	65.0 (37–77)	–	62.7 (26–87)	0.50
Body weight: mean (SD)	78.2 (36.6)	54.3 (11.7)	59.8 (12.9)	58.5 (15.4)	–	64.7 (19.9)	0.24
Creatinine (SD)	4.1 (0.5)	2.5 (2.2)	2.4 (1.5)	3.0 (3.1)	–	3.6 (2.3)	0.60

In Table, one-way analysis of variance was used to compare patient characteristics, such as sex, age, and body weight among five grades: grade 0, grade 1, grade 2, grade 3, and grade 5. There were no significant differences in sex, age, weight, and creatinine levels among five grades.

**Table 5:** Range and mean ± standard deviation of each quantitative index in uptake patterns.

Organ	Quantitative indices	Average ± S.D. (range)				*p* value
		(1) Normal (n = 54)	(2) Equal (n = 9)	(3) Invert (n = 7)	Total (n = 70)	
Liver	SUVmax	3.5 ± 1.6 (1.8–13.7)	2.7 ± 0.5 (2.2–3.8)	2.9 ± 1.2 (1.8–5.7)	3.3 ± 1.5 (1.8–13.7)	0.41
	SUVpeak	3.3 ± 1.6 (1.7–13.3)	2.5 ± 0.5 (2.0–3.5)	2.7 ± 1.1 (1.6–5.3)	3.1 ± 1.5 (1.6–13.3)	0.44
	SUVmean	2.3 ± 1.0 (1.3–8.6)	1.9 ± 0.3 (1.4–2.5)	1.8 ± 0.6 (1.2–3.3)	2.2 ± 0.9 (1.2–8.6)	0.33
Vertebrae	SUVmax	2.5 ± 1.0 (1.4–9.0)	2.6 ± 0.5 (2.0–3.6)	3.9 ± 1.4 (2.5–6.7)	2.7 ± 1.1 (1.4–9.0)	< 0.05
	SUVpeak	2.3 ± 1.0 (1.3–8.5)	2.4 ± 0.4 (1.8–3.3)	3.6 ± 1.3 (2.3–6.3)	2.5 ± 1.0 (1.3–8.5)	< 0.05
	SUVmean	1.7 ± 0.7 (1.0–5.9)	1.7 ± 0.3 (1.3–2.2)	2.5 ± 0.9 (1.5–3.9)	1.8 ± 0.7 (1.0–5.9)	< 0.05

		(1) Normal (n = 105)	(2) Equal (n = 18)	(3) Invert (n = 14)	Total (n = 137)	
Renal	SUVmax	3.0 ± 1.7 (1.3–10.9)	4.5 ± 3.5 (1.5–15.5)	3.2 ± 1.5 (1.4–6.7)	3.2 ± 2.1 (1.3–15.5)	< 0.05
	SUVpeak	2.7 ± 1.6 (1.3–10.3)	4.1 ± 3.2 (1.4–14.3)	2.9 ± 1.4 (1.3–6.2)	2.9 ± 1.9 (1.3–14.3)	< 0.05
	SUVmean (1)	1.8 ± 1.0 (0.9–7.3)	2.7 ± 2.0 (1.0–9.0)	1.9 ± 0.8 (0.9–3.6)	1.9 ± 1.2 (0.9–9.0)	< 0.05
	SUVmean (2)	1.7 ± 1.8 (0.0–9.6)	2.4 ± 2.0 (0.0–6.9)	0.9 ± 1.9 (0.0–6.7)	1.7 ± 1.9 (0.0–9.6)	0.16
	SUVmean (3)	2.1 ± 1.0 (0.0–7.8)	2.6 ± 1.2 (1.4–5.6)	1.9 ± 1.4 (0.0–4.4)	2.1 ± 1.1 (0.0–7.8)	0.19
	ANV (1)	109 ± 59 (9–431)	145 ± 91 (19–298)	180 ± 124 (52–440)	121 ± 77 (9–440)	< 0.05
	ANV (2)	24 ± 58 (0–339)	98 ± 110 (0–330)	0 ± 0 (0–1)	31 ± 70 (0–339)	< 0.05
	ANV (3)	65 ± 85 (0–400)	150 ± 143 (0–422)	40 ± 46 (0–139)	73 ± 97 (0–422)	< 0.05
	TNU (1)	222 ± 223 (36–1103)	466 ± 452 (22–1524)	425 ± 481 (53–1589)	275 ± 313 (22–1589)	< 0.05
	TNU (2)	78 ± 210 (0–1330)	397 ± 534 (0–1630)	0 ± 1 (0–3)	112 ± 290 (0–1630)	< 0.05
	TNU (3)	171 ± 272 (0–1462)	523 ± 604 (0–1872)	129 ± 192 (0–618)	213 ± 351 (0–1872)	< 0.05

In Table, one-way analysis of variance was used to compare each index, including SUVmax, SUVpeak, SUVmean, ANV, TNU, and Renal/vertebrae SUVmean among three groups: (1) Normal, (2) Equal, and (3) Invert. Three threshold-setting methods when calculating SUVmean, ANV and TNU: method (1), the threshold method (set at 40%); method (2), the constant value method (SUVmax of vertebrae); and method (3), the constant value method (SUVmean of vertebrae). There were no significant differences liver SUVmax, SUVpeak, SUVmean, and renal SUVmean (2), SUVmean (3) among the three uptake patterns. There were significant differences vertebrae SUVmax, SUVpeak, SUVmean, and renal SUVmax, SUVpeak, SUVmean (1), ANV (1), ANV (2), ANV (3), TNU (1), TNU (2), and TNU (3) among the three uptake patterns.

**Table 6:** Range and mean ± standard deviation of each quantitative index in the grade classification of normal uptake pattern.

Organ	Quantitative indices	Average ± S.D. (range)						*p* value
		Grade 0 (n = 2)	Grade 1 (n = 15)	Grade 2 (n = 9)	Grade 3 (n = 11)	Grade 4 (n = 0)	Grade 5 (n = 18)	
Liver	SUVmax	4.1 ± 1.9 (2.2–6.0)	3.3 ± 0.7 (2.2–4.9)	3.3 ± 0.4 (2.6–3.9)	3.3 ± 0.8 (1.8–4.7)		3.9 ± 2.5 (2.1–13.7)	0.79
	SUVpeak	3.7 ± 1.6 (2.0–5.3)	3.1 ± 0.7 (2.2–4.4)	3.1 ± 0.4 (2.4–3.6)	3.1 ± 0.8 (1.7–4.2)		3.6 ± 2.4 (2.0–13.3)	0.79
	SUVmean	2.3 ± 0.8 (1.5–3.1)	2.3 ± 0.6 (1.7–3.7)	2.2 ± 0.3 (1.9–2.7)	2.2 ± 0.5 (1.4–3.0)		2.6 ± 1.5 (1.3–8.6)	0.86
Vertebrae	SUVmax	2.7 ± 0.5 (2.2–3.3)	2.6 ± 0.6 (1.7–3.5)	2.3 ± 0.2 (2.0–2.8)	2.1 ± 0.4 (1.4–2.7)		2.8 ± 1.6 (1.4–9.0)	0.47
	SUVpeak	2.5 ± 0.4 (2.0–2.9)	2.4 ± 0.5 (1.7–3.3)	2.2 ± 0.2 (1.8–2.7)	2.0 ± 0.4 (1.4–2.5)		2.6 ± 1.5 (1.3–8.5)	0.48
	SUVmean	1.7 ± 0.2 (1.5–1.9)	1.7 ± 0.4 (1.1–2.3)	1.5 ± 0.2 (1.3–1.9)	1.4 ± 0.2 (1.0–1.9)		1.9 ± 1.0 (1.0–5.9)	0.40

		Grade 0 (n = 3)	Grade 1 (n = 29)	Grade 2 (n = 17)	Grade 3 (n = 22)	Grade 4 (n = 0)	Grade 5 (n = 34)	
Renal	SUVmax	2.1 ± 0.2 (1.9–2.3)	2.0 ± 0.4 (1.3–2.9)	2.2 ± 0.3 (1.6–2.9)	2.6 ± 0.6 (1.6–3.8)		4.5 ± 2.3 (2.1–10.9)	< 0.05
	SUVpeak	1.9 ± 0.1 (1.7–2.1)	1.9 ± 0.4 (1.3–2.6)	2.0 ± 0.3 (1.5–2.7)	2.4 ± 0.6 (1.5–3.5)		4.1 ± 2.1 (2.0–10.3)	< 0.05
	SUVmean (1)	1.2 ± 0.0 (1.2–1.2)	1.2 ± 0.2 (0.9–1.7)	1.3 ± 0.2 (1.0–1.9)	1.6 ± 0.3 (1.2–2.1)		2.7 ± 1.4 (1.3–7.3)	< 0.05
	SUVmean (2)	0.0 ± 0.0 (0.0–0.0)	0.1 ± 0.5 (0.0–1.9)	1.0 ± 1.2 (0.0–2.8)	2.2 ± 0.6 (0.0–2.9)		3.1 ± 1.9 (0.0–9.6)	< 0.05
	SUVmean (3)	1.9 ± 0.2 (1.6–2.0)	1.7 ± 0.5 (0.0–2.4)	1.7 ± 0.2 (1.5–2.1)	1.8 ± 0.3 (1.2–2.3)		2.7 ± 1.4 (1.6–7.8)	< 0.05
	ANV (1)	99 ± 12 (82–109)	83 ± 31 (25–140)	106 ± 39 (45–172)	90 ± 58 (9–263)		146 ± 69 (35–431)	< 0.05
	ANV (2)	0 ± 0 (0–0)	0 ± 0 (0–1)	0 ± 0 (0–1)	10 ± 11 (0–38)		68 ± 86 (0–339)	< 0.05
	ANV (3)	3 ± 3 (0–6)	5 ± 7 (0–29)	27 ± 16 (4–78)	61 ± 45 (3–215)		142 ± 104 (9–400)	< 0.05
	TNU (1)	118 ± 15 (98–132)	99 ± 38 (36–199)	142 ± 55 (64–235)	161 ± 121 (42–544)		416 ± 290 (58–1103)	< 0.05
	TNU (2)	0 ± 0 (0–0)	0 ± 0 (0–2)	0 ± 1 (0–2)	24 ± 28 (0–105)		225 ± 321 (0–1330)	< 0.05
	TNU (3)	4 ± 4 (1–10)	8 ± 10 (0–43)	46 ± 26 (5–120)	126 ± 106 (24–467)		418 ± 356 (19–1462)	< 0.05
Renal/vertebrae	Renal SUVmean (1)/vertebrae SUVmean	0.7 ± 0.1 (0.6–0.8)	0.7 ± 0.2 (0.0–0.9)	0.9 ± 0.1 (0.8–1.1)	1.2 ± 0.1 (1.0–1.3)		1.5 ± 0.7 (0.9–3.7)	< 0.05
	Renal SUVmean (2)/vertebrae SUV mean	0.0 ± 0.0 (0.0–0.0)	0.1 ± 0.4 (0.0–1.5)	0.9 ± 0.8 (0.0–1.7)	1.6 ± 0.1 (1.5–1.7)		1.6 ± 0.7 (0.0–3.2)	< 0.05
	Renal SUVmean (3)/vertebrae SUVmean	1.1 ± 0.0 (1.1–1.1)	1.0 ± 0.3 (0.0–1.2)	1.1 ± 0.0 (1.1–1.2)	1.3 ± 0.1 (1.2–1.4)		1.5 ± 0.4 (1.1–2.9)	< 0.05

In Table, one-way analysis of variance was used to compare each index, including SUVmax, SUVpeak, SUVmean, ANV, TNU, and renal/vertebrae SUVmean among five grades: grade 0, grade 1, grade 2, grade 3, and grade 5. Three threshold-setting methods when calculating SUVmean, ANV and TNU: method (1), the threshold method (set at 40%); method (2), the constant value method (SUVmax of vertebrae); and method (3), the constant value method (SUVmean of vertebrae). There were no significant differences in liver and vertebrae SUVmax, SUVpeak, and SUVmean among the five grades. There were significant differences in all renal quantitative indices and renal SUVmean/vertebrae SUVmean among the five grades.

**Table 7:**
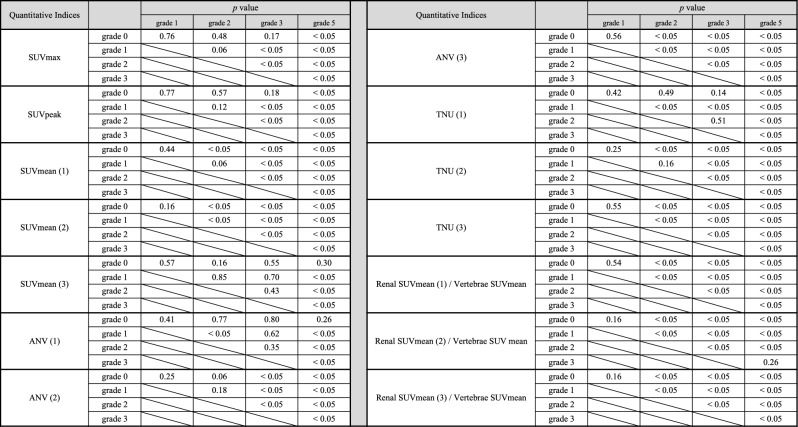
Results of significant differences between each set of two grades in each quantitative index.

In Table, after F test, Student’s t-test or Welch’s t test was used to compare between each two grades. Each set of two grades showed statistically significant differences except for grade 0, 1, 2, and 3, and grade 1 and 2 in SUVmax and SUVpeak; grade 0 and 1 and grade 1 and 2 in SUVmean (1); grade 0 and 1 in SUVmean (2); grade 0, 1, 2, 3, and 5, grade 1, 2, and 3, and grade 2 and 3 in SUVmean (3); grade 0, 1, 2, 3, and 5, grade 1 and 3, and grade 2 and 3 in ANV (1); grade 0, 1, and 2 and grade 1 and 2 in ANV (2); grade 0 and 1 in ANV (3); grade 0, 1, 2, and 3, and grade 2 and 3 in TNU (1); grade 0 and 1, grade 1 and 2 in TNU (2); grade 0 and 1 in TNU (3); grade 0 and 1 in SUVmean (1)/vertebrae SUVmean; grade 0 and 1 and grade 3 and 5 in SUVmean (2)/vertebrae SUVmean; and grade 0 and 1 in SUVmean (3)/vertebrae SUVmean.

**Table 8:** Range and mean ± standard deviation of each quantitative index in interstitial nephritis (IN) and non-IN.

Organ	Quantitative indices	Average ± S.D. (range)		
		IN, IN + other disease (n = 36)	IN only (n = 26)	Non-IN (n = 29)
Liver	SUVmax	3.5 ± 1.9 (2.1–13.7)	3.6 ± 2.2 (2.1–13.7)	3.3 ± 1.1 (1.8–6.0)
	SUVpeak	3.3 ± 1.8 (2.0–13.3)	3.4 ± 2.1 (2.0–13.3)	3.0 ± 1.0 (1.6–5.3)
	SUVmean	2.3 ± 1.2 (1.3–8.6)	2.4 ± 1.3 (1.4–8.6)	2.2 ± 0.7 (1.2–3.7)
Vertebrae	SUVmax	2.7 ± 1.2 (1.4–9.0)	2.8 ± 1.4 (1.6–9.0)	2.7 ± 1.1 (1.4–6.7)
	SUVpeak	2.5 ± 1.1 (1.3–8.5)	2.6 ± 1.3 (1.5–8.5)	2.5 ± 1.0 (1.4–6.3)
	SUVmean	1.8 ± 0.8 (1.0–5.9)	1.9 ± 0.9 (1.2–5.9)	1.8 ± 0.7 (1.0–3.9)

		IN, IN + other disease (n = 71)	IN only (n = 51)	Non-IN (n = 57)
Renal	SUVmax	4.0 ± 2.5* (1.6–15.5)	4.2 ± 2.9* (1.6–15.5)	2.4 ± 1.0 (1.4–6.7)
	SUVpeak	3.7 ± 2.3* (1.5–14.3)	3.9 ± 2.7 * (1.5–14.3)	2.2 ± 0.9 (1.3–6.2)
	SUVmean (1)	2.4 ± 1.5* (1.1–9.0)	2.6 ± 1.7* (1.1–9.0)	1.5 ± 0.5 (0.9–3.6)
	SUVmean (2)	2.5 ± 1.9* (0.0–9.6)	2.6 ± 2.1* (0.0–9.6)	0.9 ± 1.4 (0.0–6.7)
	SUVmean (3)	2.5 ± 1.2* (0.0–7.8)	2.6 ± 1.4* (0.0–7.8)	1.7 ± 0.8 (0.0–4.4)
	ANV (1)	137 ± 75 (9–431)	138 ± 79 (19–431)	108 ± 80 (25–440)
	ANV (2)	58 ± 90* (0–339)	69 ± 102* (0–339)	3 ± 8 (0–38)
	ANV (3)	117 ± 114* (0–422)	122 ± 127* (0–422)	28 ± 41 (0–215)
	TNU (1)	365 ± 330* (22–1524)	394 ± 373* (22–1524)	192 ± 283 (36–1589)
	TNU (2)	209 ± 380* (0–1630)	257 ± 436* (0–1630)	7 ± 21 (0–105)
	TNU (3)	353 ± 432* (0–1872)	395 ± 490* (0–1872)	70 ± 129 (0–618)
Renal/vertebrae	Renal SUVmean (1)/vertebrae SUVmean	1.4 ± 0.7* (0.6–4.2)	1.4 ± 0.8* (0.6–4.2)	0.9 ± 0.2 (0.5–1.4)
	Renal SUVmean (2)/vertebrae SUV mean	1.4 ± 0.9* (0.0–3.2)	1.4 ± 0.9* (0.0–3.2)	0.6 ± 0.8 (0.0–1.8)
	Renal SUVmean (3)/vertebrae SUVmean	1.4 ± 0.4* (0.0–2.9)	1.4 ± 0.5* (0.0–2.9)	1.0 ± 0.3 (0.0–1.4)

In Table, after F test, Student’s t-test or Welch’s t test was used to compare between each set of two groups: IN, IN + other diseases—non-IN, IN only—non-IN. Three threshold-setting methods when calculating SUVmean, ANV and TNU: method (1): the threshold method (set at 40%); method (2), the constant value method (SUVmax of vertebrae); and method (3), the constant value method (SUVmean of vertebrae). Thirteen indices were higher in the IN group than in the non-IN group, except for liver, vertebrae, and ANV (1).

*p < 0.05 in comparison to non-IN.

## Results

### Phantom study

BCF was obtained as 3249.22 [Bq/cps].

### Patient study

As summarized in Table 2, there were no significant differences in sex, age, weight, and creatinine levels between IN and non-IN subjects.

### Visual evaluation

Two nuclear medicine specialists independently interpreted the planar and SPECT images. The issue of the same images being graded differently by the specialists was resolved by consensus to provide a final grade. Although liver uptake is usually higher than vertebral body uptake, several cases showed uptake wherein the liver and vertebral body uptakes were equal or vertebral body uptake was higher than liver uptake. Figure 1 shows examples of three uptake patterns: (1) Normal, (2) Equal, (3) Invert. The results of the classification into three groups according to the uptake pattern are presented in Table 3; there were no significant differences in sex, age, weight, and creatinine levels among the three uptake patterns. The results of the 6-score grading of renal uptake are presented in Table 4; there were no significant differences in sex, age, weight, and creatinine levels among the five grades.

### Quantitative indices

As shown in Fig. 2, the liver VOI was set to be as wide as possible and to not include other organs. The vertebral body VOI was set to two vertebral bodies at the same level as the kidneys. The kidney VOI was set separately for the left and right sides; the right kidney VOI was especially set so as not to include the liver.

In the grade 2 example shown in the top row of Fig. 3, in method (1), or the threshold method (set at 40%), the VOI was set wider than the actual renal uptake; in method (2), or the constant value method (SUVmax of vertebrae), the VOI was set narrower than the actual renal uptake, and in method (3), or the constant value method (SUVmean of vertebrae), the VOI matched the actual renal uptake.

The 17 quantitative indices of the uptake patterns are listed in Table 5. Twelve indices were significantly different among the three groups, except for liver indices, renal SUV_mean_ (2), and renal SUV_mean_ (3). Figure 4 compares the SUV of the liver and vertebrae of each patient for each uptake pattern. In (1) the Normal uptake pattern, liver SUV was significantly higher than vertebrae SUV. In (2) the Equal uptake pattern, there was no significant difference between liver SUVmax and SUVpeak and vertebrae SUVmax and SUVpeak; liver SUVmean was significantly higher than vertebrae SUVmean. In (3) the Invert uptake pattern, vertebrae SUV was significantly higher than liver SUV.

The 20 quantitative indices used for the grade classification are listed in Table 6. Fourteen indices were significantly different among the five grades, except for the liver and vertebral indices. Figure 5 presents the renal quantitative indices for each grade of the normal uptake pattern. The statistical analysis for Fig. 5 is presented in Table 7. Each set of two grades showed statistically significant differences except for grade 0, 1, 2, and 3, and grade 1 and 2 in SUVmax and SUVpeak; grade 0 and 1 and grade 1 and 2 in SUVmean (1); grade 0 and 1 in SUVmean (2); grade 0, 1, 2, 3, and 5, grade 1, 2, and 3, and grade 2 and 3 in SUVmean (3); grade 0, 1, 2, 3, and 5, grade 1 and 3, and grade 2 and 3 in ANV (1); grade 0, 1, and 2 and grade 1 and 2 in ANV (2); grade 0 and 1 in ANV (3); grade 0, 1, 2, and 3, and grade 2 and 3 in TNU (1); grade 0 and 1, grade 1 and 2 in TNU (2); grade 0 and 1 in TNU (3); grade 0 and 1 in SUVmean (1)/vertebrae SUVmean; grade 0 and 1 and grade 3 and 5 in SUVmean (2)/vertebrae SUVmean; and grade 0 and 1 in SUVmean (3)/vertebrae SUVmean.

**Figure 7:**
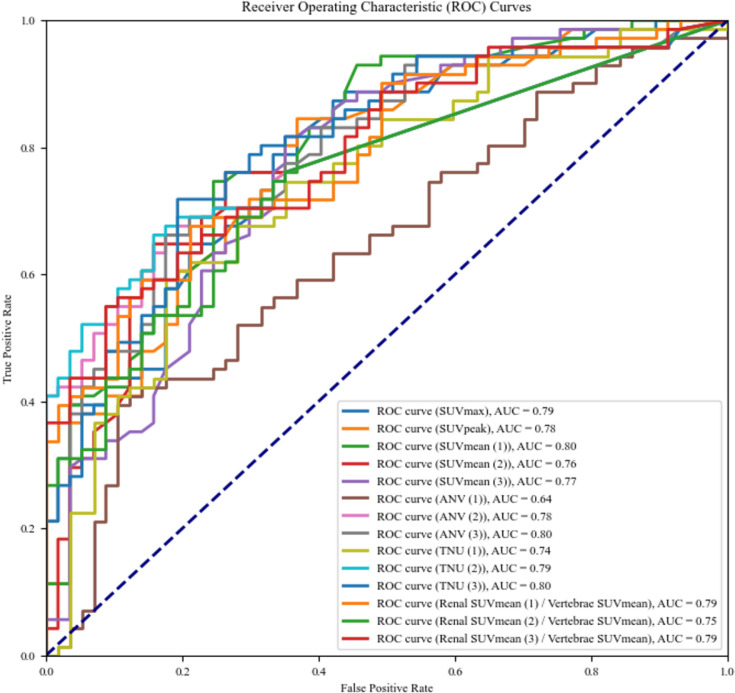
ROC curve analysis for quantitative index. IN groups (interstitial nephritis, interstitial nephritis + other diseases) (n = 36) vs non-IN groups (n = 29). SUV_mean_ (1), ANV (3), and TNU (3) showed the highest AUC (0.80).

**Figure 8:**
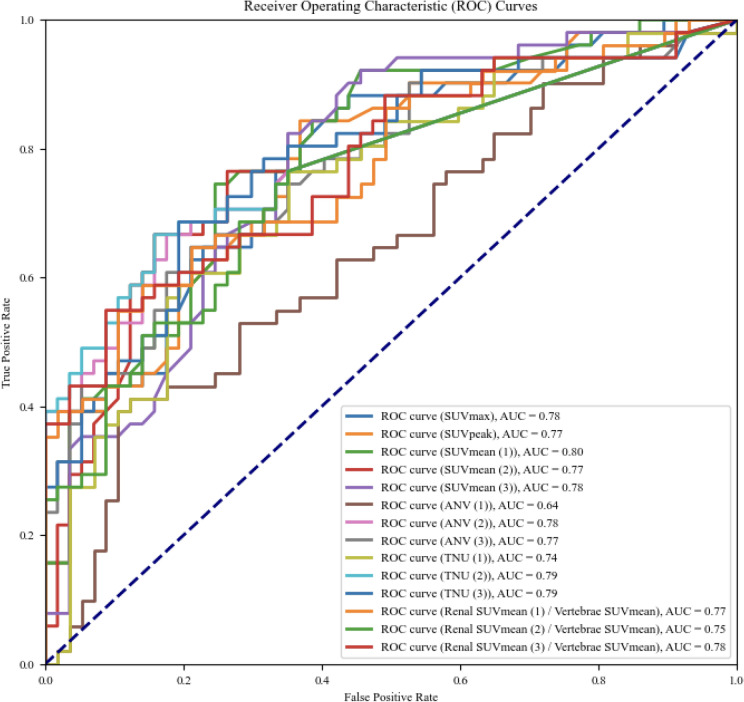
ROC curve analysis for each quantitative index. IN (n = 26) vs non-IN groups (n = 29). SUV_mean_ (1) had the highest AUC (0.80).

The 20 quantitative indices used for differentiating IN and non-IN participants are listed in Table 8. Thirteen indices were higher in the IN group (*p* < 0.05) than in the non-IN group, except for liver, vertebrae, and ANV (1). Figure 6 shows each quantitative indicator for the IN and non-IN groups. There were significant differences between the IN groups and non-IN groups in all quantitative indicators, except ANV (1). The statistical results of the receiver operating curve (ROC) analysis for the differentiation of the IN group (n = 36) from the non-IN group (n = 29) for each quantitative index are shown in Fig. 7 and Table 9. SUV_mean_ (1), ANV (3), and TNU (3) showed the highest AUC (0.80). The statistical results of the ROC analysis for the differentiation of IN (n = 26) from non-IN groups (n = 29) for each quantitative index are shown in Fig. 8 and Table 10. SUV_mean_ (1) had the highest AUC (0.80).

**Table 9:** ROC analysis (interstitial nephritis (IN) groups (IN, IN + other diseases) (n = 36) vs non-IN groups (n = 29)).

Quantitative indices	Sensitivity (%)	Specificity (%)	AUC	Cut-off value
SUVmax	79	67	0.79	2.4
SUVpeak	85	63	0.78	2.1
SUVmean (1)	75	75	0.80	1.6
SUVmean (2)	76	74	0.76	1.9
SUVmean (3)	82	65	0.77	1.8
ANV (1)	39	89	0.64	159
ANV (2)	66	82	0.78	1
ANV (3)	66	82	0.80	40
TNU (1)	59	82	0.74	202
TNU (2)	66	84	0.79	3
TNU (3)	72	81	0.80	62
Renal SUVmean (1)/vertebrae SUVmean	68	79	0.79	1.0
Renal SUVmean (2)/vertebrae SUVmean	75	67	0.75	1.4
Renal SUVmean (3)/vertebrae SUVmean	55	91	0.79	1.3

Three threshold-setting methods when calculating SUVmean, ANV and TNU: method (1), the threshold method (set at 40%); method (2), the constant value method (SUVmax of vertebrae); and method (3), the constant value method (SUVmean of vertebrae).

**Table 10:** ROC analysis (interstitial nephritis (IN) only (n = 26) vs non-IN groups (n = 29)).

Quantitative indices	Sensitivity (%)	Specificity (%)	AUC	Cut-off value
SUVmax	88	56	0.78	2.2
SUVpeak	84	63	0.77	2.1
SUVmean (1)	75	75	0.80	1.6
SUVmean (2)	67	84	0.77	2.3
SUVmean (3)	82	65	0.78	1.8
ANV (1)	41	88	0.64	154
ANV (2)	67	82	0.78	1
ANV (3)	65	79	0.77	36
TNU (1)	76	65	0.74	136
TNU (2)	67	84	0.79	3
TNU (3)	69	81	0.79	62
Renal SUVmean (1)/vertebrae SUVmean	59	86	0.77	1.1
Renal SUVmean (2)/vertebrae SUVmean	76	65	0.75	1.3
Renal SUVmean (3)/vertebrae SUVmean	55	91	0.78	1.3

Three threshold-setting methods when calculating SUVmean, ANV and TNU: method (1), the threshold method (set at 40%); method (2), the constant value method (SUVmax of vertebrae); and method (3), the constant value method (SUVmean of vertebrae).

## Discussion

This study demonstrates the relationship between conventional visual assessment and new Ga-S-based quantitative indices and clarifies the utility of the new indices in the management of nephritis.

^67^Ga is excreted mainly from the kidneys within 24 h after intravenous administration, with the kidneys showing the highest accumulation within 24 h. Even though approximately 12% of the administered dose is excreted from the kidney, the liver is the main route of excretion from 48 to 72 h, and high accumulation is seen in the bone, liver, and spleen. About 1/3 of the dose is excreted within 1 week of administration, with the remaining 2/3 remaining in the liver (6%), spleen (1%), kidney (2%), bone and bone marrow (24%), and other soft tissues (34%). Relatively high accumulation was also observed in the intestinal tract even after excretion through the liver. Although the mechanism of accumulation of ^67^Ga-citrate in tumors or inflamed lesions has not yet been fully elucidated, several accumulation processes have been suggested. In tumors, ^67^Ga-citrate administered into the veins binds to transferrin in the serum to form a transferrin-^67^Ga complex, which acts on the transferrin receptor of the tumor cells and is taken up into the cells. Intracellularly, it is distributed in the cytoplasm, including lysosomes, as ^67^Ga-ferritin, but most of it is transported to the microvesicles and endoplasmic reticulum, where it binds to macromolecular proteins essential for tumor cell function. For inflammatory lesions, several mechanisms have been postulated as follows: (1) Increased blood flow: the ionic form may enter cells due to inflammation-induced enlargement of small arteries and increased permeability of capillaries. (2) Leukocyte uptake: The uptake of ^67^Ga by human polymorphonuclear leukocytes was higher than that by lymphocytes, suggesting that ^67^Ga binds to the membrane surfaces of polymorphonuclear leukocytes. (3) Lactoferrin binding: ^67^Ga binds to lactoferrin, which is abundant in neutrophils, and accumulates on neutrophils at inflammatory sites. In addition, the mechanisms of Ga accumulation in inflamed tissues have been reported to include increased vascular permeability at the site of inflammation and the presence of acidic mucopolysaccharides in the inflamed tissues^13–16^. Abnormal renal uptake can be mainly observed in inflammatory diseases such as interstitial nephritis, renal tumors such as renal cell carcinoma, and renal failure due to delayed gallium excretion from the kidney^17^. This study focused on the utility of Ga-S in the management of nephritis.

IN, which is characterized by inflammatory infiltration into the renal interstitium, is a cause of renal failure. It can be acute or chronic. The main causes include drugs, infectious diseases, immune diseases, and tubulointerstitial nephritis^18^. The WHO classification of tubulointerstitial diseases into detailed categories is based on underlying diseases and causes^19^.

Renal biopsy is the gold standard for diagnosing IN; however, patients may be contraindicated for biopsy or reluctant to undergo the test owing to its invasive nature. Ga-S scanning has been widely used for many years to study numerous renal inflammatory diseases^20^. Studies in rats have demonstrated that ^67^Ga-citrate scintigraphy is highly accurate in differentiating experimentally induced acute interstitial nephritis (AIN) from drug-induced acute tubular necrosis (ATN) in normal rat kidneys^21^. Some reports have suggested that ^67^Ga-citrate scintigraphy may be a useful tool for diagnosing IN^3,22–24^. However, a study of 12 patients with ^67^Ga-citrate scintigraphy who were diagnosed with noninfectious interstitial nephritis by renal biopsy reported a sensitivity of 58%^6^. ^67^Ga-citrate scintigraphy is a noninvasive diagnostic method that can help diagnose AIN when a renal biopsy is contraindicated or refused by the patient. However, the limitations of this test should be recognized prior to its use in these patients^25^. Ga-S showed physiological accumulation in the liver, spine, soft tissues, and lacrimal glands, with the highest accumulation in the liver. Traditionally, when evaluating renal accumulation by using gallium scintigraphy, visual grading is performed using the liver and spine as references. Liver accumulation of gallium is often uniform, but accumulation in the spine is heterogeneous due to deformational and degenerative spondylitis and often shows multinodular hyperaccumulation, which is more evident on SPECT than on planar images. Visual grading depends on the diagnostic ability of the observer and is subjective and less objective. Therefore, novel quantitative and objective indicators must be developed. Therefore, if we use quantitative evaluation using the proposed SUV, it can be evaluated regardless of the accumulation in the liver or vertebral body; therefore, it can be evaluated even in cases such as the above, and objective evaluation is possible. In addition, understanding disease progression and predicting the effects of steroids by numerically evaluating interstitial nephritis may be extremely useful in its management. Based on these results, we believe that quantifying Ga-S is important.

This uptake mechanism is thought to be due to the binding of ^67^Ga-citrate administered into the blood to transferrin in the serum^26^. ^67^Ga-citrate scintigraphy is usually considered positive when renal uptake is equal to or higher than liver or spine uptake^5,6^. However, ^67^Ga-citrate is typically taken up by the liver and spine and may be influenced by transferrin concentration^27^. Some cases showed different uptake patterns from normal, such as higher vertebral than liver uptake, and were classified into three groups (Fig. 1). As shown in Fig. 4 and Table 5, in the case of (2)-equal class, abnormal uptake patterns were visually equal for liver and vertebral uptake, and the SUVs of the liver and vertebrae were almost equal when compared. In the (3)-invert class, the abnormal uptake pattern was visually higher for vertebral than for liver uptake, and the SUVs of the liver and vertebrae were higher than those of the liver. In summary, the visual appearance of an anomalous uptake pattern was consistent with the quantitative SUV results, suggesting the presence of an anomalous uptake pattern. In the (3)-invert group, the SUV of the liver was lower and that of the vertebral body was higher than in the (1)-normal group, suggesting that the liver, vertebral body, or transferrin effects may be the cause of the abnormal uptake pattern. Furthermore, Table 3 shows that there was no difference in creatinine levels depending on the accumulation pattern, suggesting that renal function may not be related to accumulation in the liver or vertebral body. In the (3)-invert class, ANV (2) with vertebral SUV_max_ as the threshold value was 0. This may be due to the vertebral SUVs being higher than the normal uptake pattern, and the vertebral SUV_max_ being higher than the renal SUVs. Since visual evaluation assumes that liver uptake is higher than vertebral uptake, conventional visual evaluation cannot be applied to abnormal uptake patterns such as (2) and (3); however, the fact that the quantitative index-based evaluation in the present study can evaluate ^67^Ga-citrate uptake even in abnormal uptake patterns is considered a major advantage. Additionally, when uptake in the vertebral body or liver is patchy and high uptake is observed in some areas, visual evaluation is often confusing; however, the quantitative evaluation in this study is expected to resolve this issue.

As shown in Fig. 5 and Table 6, focusing on the relationship between each quantitative index of the renal gland and the grade by visual assessment, each quantitative index tended to show higher values as the grade increased. This indicates that the results of grade classification by visual evaluation and quantitative evaluation by each quantitative index were consistent, and we believe that grade classification using quantitative indices can be performed.

As shown in Figs. 3 and 5, using the vertebral SUVmax as a threshold, there were several cases of visual renal uptake of grades 1–5 with zero values for each quantitative index, which could lead to false negatives and the underestimation of uptake. The method of using 40% as the threshold when 100% of the maximum value is within the VOI, which is the default setting of the analysis software, may lead to the inclusion of areas of low uptake, and there is a possibility of overestimation. The method of using the vertebral SUV_mean_ as a threshold may be considered appropriate as a positive result is defined as an uptake that is visually equal to or higher than that of the vertebrae; however, this method is considered useful only for normal uptake patterns. ^67^Ga-citrate scintigraphy is usually considered positive if posterior planar imaging demonstrates renal uptake equal to or higher than that of the vertebrae^6^. We also calculated the SUV of the kidney divided by the SUV of the vertebral body. The mean value for grade 2 renal uptake was equivalent to that of the vertebral body and was 0.9 or close to 1, with values below 1 for grade 1 and below and above 1 for grades 3 and above. Although it is difficult to determine positive or negative renal SUV alone, using this index, a value greater than 1 is considered positive, and the criteria for judgment are considered easy to understand. However, this indicator is useful only for normal uptake patterns; abnormal uptake patterns (vertebral uptake higher than normal) may lead to underestimation.

In Fig. 6 and Table 8, values of all quantitative indicators of IN, except ANV (1), were significantly higher than those of non-IN. However, some IN cases had quantitative index values of 0, and some non-IN cases had high quantitative index values. Thus, although the quantitative index may be useful in differentiating between IN and non-IN, it is limited because the ^67^Ga-citrate uptake mechanism is complex and unclear and there are diseases other than IN wherein renal uptake is observed.

In the ROC analysis (Fig. 7, Table 9), SUV_mean_ (1), ANV (3), and TNU (3) showed good results, with an AUC of 0.80. Threshold setting (1) is independent of vertebral uptake and can be applied to all uptake patterns; however, the AUC = 0.64 for ANV (1) was not good, and using threshold setting (1) as a threshold for ANV may lead to overestimation. Since threshold settings (2) and (3) use vertebral SUV as the threshold, uptake patterns (2) and (3) may lead to an underestimation of uptake. Therefore, we believe that SUV_mean_ (1), which can be applied to any uptake pattern, is useful for evaluating the degree of ^67^Ga-citrate uptake, and that ANV (3) and TNU (3) are useful for evaluating the volume. However, caution should be exercised when using the vertebral SUV as the threshold value.

In the ROC analysis (Fig. 8, Table 10), SUV_mean_ (1) showed good results (AUC = 0.80). Therefore, when focusing on distinguishing between IN and non-IN, the SUV_mean_ (1) was considered the most useful quantitative indicator. In addition, quantitative indicators, such as SUV, may be useful in determining the effectiveness of treatment and monitoring the progress of renal inflammation. Furthermore, from the results in Tables 8 and 9, there was no significant difference in the AUC between IN only and IN+ other diseases, suggesting that the presence of other diseases may not have a large effect on the diagnosis of IN.

Mimiko et al. proposed the RU as a semi-quantitative index for evaluating IN in ^67^Ga-citrate. RU was calculated by setting the left kidney and background ROIs, as well as calculating the “kidney/soft tissue ratio” from the average count per pixel^4^. Since this method uses planar images, it has the advantage of being simple and short in imaging time; however, its calculation is a count ratio, and it cannot obtain information on the SUV or volume. Despite that, our proposed method uses SPECT/CT imaging, which has the disadvantages of increased imaging time and radiation exposure. It is possible to set the region of interest only to the kidney, which eliminates the effects of overlapping organs. A major advantage of this method is that ^67^Ga-citrate uptake can be evaluated based on SUV and volume.

Every institution with a SPECT/CT scanner can assess this method with reference to the original BCF of the gamma camera and with the introduction of analytical software. The CT data of SPECT/CT were used for attenuation correction and morphological information. By using CT, gallium excreted in the colon, as well as accumulated in the kidney and liver, could be confirmed. In the kidneys, it is also possible to evaluate whether the accumulation was predominantly cortical or medullary and to confirm that the nodular accumulation in the spine was consistent with degenerative spondylosis.

This study has two limitations. One is the bias of the used VOI settings. The threshold used in this study was the default value of the analysis software, and other threshold values were not considered. Therefore, there is room for discussion regarding the diagnostic performance of different threshold values. Second, there is the potential foe patient selection bias because this was a single-center study, and the number of patients was not large. A multicenter study with a larger population is necessary to confirm the utility of the new ANV and TNU indices with GI-BONE with reference to the original BCF of each institution. Calibration of gamma camera systems is also necessary to normalize and standardize the method. In addition, there are many known mechanisms of gallium accumulation, ranging from physiological to pathological, and some mechanisms are still unknown. The non-IN group includes many kinds of diseases. Owing to the diversity of patients and mechanisms of gallium accumulation, there may be diseases and mechanisms that present similar quantitative indices, which is also controversial. Recent advances in radiomics analysis using medical imaging can be applied to gallium scintigraphy, and the extracted features may be useful in differentiating renal diseases, assessing the severity of renal damage, and predicting prognosis^28,29^.

In conclusion, we attempted to quantify the renal uptake of ^67^Ga-citrate in patients with abnormal renal uptake using SPECT/CT and proposed new Ga-S-based biomarkers. As the visual evaluation grade increased, the values of each quantitative index also tended to increase. SUV_mean_ (1), ANV (3), and TNU (3) were useful quantitative indices for evaluating renal inflammation, and SUV_mean_ (1) was useful as a quantitative index to distinguish between IN and non-IN. Additionally, these new quantitative indicators enable the evaluation of abnormal uptake patterns that are difficult to assess visually.

## Data Availability

The data that support the findings of this study are not openly available due to reasons of sensitivity and are available from the corresponding author upon reasonable request. Data are located in controlled access data storage at Hospital Information System of Tokushima University Hospital.
